# Expression of bioactive bone morphogenetic proteins in the subacromial bursa of patients with chronic degeneration of the rotator cuff

**DOI:** 10.1186/ar1965

**Published:** 2006-05-23

**Authors:** Jana Neuwirth, Renée AE Fuhrmann, Amanda Veit, Matthias Aurich, Ilmars Stonâns, Tilo Trommer, Peter Hortschansky, Susanna Chubinskaya, Juergen A Mollenhauer

**Affiliations:** 1 Department of Orthopedics, University of Jena, Klosterlausnitzerstr. 81, D-07607 Eisenberg; 2 Leibniz Institute for Natural Product Research and Infection Biology, Hans-Knöll Institute, Beutenbergstr. 11a, D-07745 Jena; 3 Department of Biochemistry, Rush University Medical Center, 1653 W. Congress Parkway, Chicago, IL 60612, USA

## Abstract

Degeneration of the rotator cuff is often associated with inflammation of the subacromial bursa and focal mineralization of the supraspinatus tendon. Portions of the supraspinatus tendon distant from the insertion site could transform into fibrous cartilage, causing rotator-cuff tears owing to mechanical instability. Indirect evidence is presented to link this pathology to ectopic production and secretion of bioactive bone morphogenetic proteins (BMPs) from sites within the subacromial bursa. Surgically removed specimens of subacromial bursa tissue from patients with chronic tears of the rotator cuff were analyzed by immunohistochemistry and reverse transcription-PCR. Bioactive BMP was detected in bursa extracts by a bioassay based on induction of alkaline phosphatase in the osteogenic/myogenic cell line C2C12. Topical and differential expression of BMP-2/4 and BMP-7 mRNA and protein was found in bursa tissue. The bioassay of C2C12 cells revealed amounts of active BMP high enough to induce osteogenic cell types, and blocking BMP with specific antibodies or soluble BMP receptors Alk-3 and Alk-6 abolished the inductive properties of the extract. Sufficient information was gathered to explain how ectopic expression of BMP might induce tissue transformation into ectopic bone/cartilage and, therefore, promote structural degeneration of the rotator cuff. Early surgical removal of the subacromial bursa might present an option to interrupt disease progression.

## Introduction

Alterations to the rotator cuff owing to chronic degenerative and traumatic lesions are not only frequently diagnosed in the elderly, but are also seen as a result of occupational or sports activities. It is also widely accepted that cuff ruptures attributed to an accident mostly occur in patients suffering from asymptomatic degenerative lesions of the tendon.

Chronic degeneration of the rotator cuff frequently presents as an 'impingement syndrome', with painful restriction of joint mobility. X-rays can reveal dense clouds within the tendon, establishing the diagnosis of 'tendinosis calcarea' as an additional pathologic trait. Ultrasound or magnetic resonance imaging usually demonstrates an enlarged subacromial bursa adjacent to the rotator-cuff lesion. Surgical treatment focuses on anatomic and functional reconstruction. Nevertheless, the results are often poor. Even after debridement of the torn tendon, followed by suturing or osseous reattachment, the tissue does not better: does not re-adjust to the functional and mechanical demands. As a result, nearly two-thirds of surgically reconstructed rotator cuffs experience a re-rupture [1,2]. This clinical dilemma emphasizes that the underlying pathomechanisms involving the rotator-cuff tendon and subacromial bursa are not yet understood.

One of the pathologic hallmarks is inflammation, with consequent hypertrophy of the subacromial bursa [3,4]. Hypertrophy of the bursa is regarded as one of the causes of the chondroid transformation of the supraspinatus tendon because the expanded bursa tissue can act on the tendon and surrounding tissues. Expansion of the bursa, however, does not explain calcification of the tendon. Therefore, tendinosis calcarea is classified as a separate disease entity, although quite frequently co-existing with an impingement syndrome attributable to rotator-cuff tears [5-8].

Here, we present evidence that chronic degeneration of the rotator cuff is associated with inflammation-related induction of bone morphogenetic protein (BMP) activity in the joint. The deposition and activation of significant quantities of BMP could, in part, explain the observed rotator-cuff pathologies, together with inflammation-induced proliferation of connective tissue. BMP might induce differentiation of competent cell types, such as mesenchymal precursors, tendon cells, and other soft tissue cells, into osteochondral lineages, to form ectopic populations of (fibro-) cartilage and mineralized tissues. To support this hypothesis, experiments were carried out to define the level of BMP and its activity within the affected subacromial bursa.

## Materials and methods

### Tissue

Subacromial bursa was harvested from a total of 29 patients (age range, 36–75 years; median age, 57 ± 9.8 years; normal distribution (Kolmogorov-Smirnov) during surgical interventions to address rotator-cuff tears, with patient consent and institutional approval (approval #0981-10/02 from the Ethics Committee of the Medical Faculty at the University of Jena, Jena, Germany). Two patients were acute trauma cases who received emergency shoulder surgery and served as donors of 'normal' bursa tissue. The tissue was collected in an oriented fashion and immediately frozen using dry ice. Aliquots from segments close to the acromion, the medial portion, the basal portion, and close to the tendon (containing tendineus tissue) were excised using a 2.5-mm tissue punch, placed into an RNA extraction solution (TRIzol™ reagent; Life Technologies, Invitrogen, Carlsbad, CA, USA), and stored at -80°C. The deep-frozen tissue was also stored at -80°C before use.

### Bone morphogenetic protein extraction

Deep-frozen tissue was placed into 4 M guanidinium/HCl (approximately 1 ml/100 mg tissue), which was supplemented with protease inhibitors (0.1 mM phenylmethanesulfonylfluoride, 0.1 mM N-ethylmaleinimide, and 5 mM ethylendiaminetetraacetic acid). Extraction was performed overnight at 4°C. The enrichment of BMP followed established procedures. The extract was centrifuged at 10,000 rpm in a microvial centrifuge and then exhaustively dialyzed against column buffer (0.05 M Na-acetate and 30% isopropanol; pH 5.0). The clear solution was placed on top of a small bed of MonoS beads (GE Healthcare, Munich, Germany) in a 10 ml syringe and allowed to flow by gravity. The beads were washed with column buffer, and the bound protein was stepwise eluted with 0.1 M, 0.5 M, and 1 M NaCl in column buffer. Typically, most of the BMP activity was detected in the 0.5 M NaCl fraction. The eluates were concentrated by lyophilization and dialyzed against PBS before being applied to the cell culture.

### Cell culture

The osteogenic mouse precursor cell line C2C12 (purchase number CRL-1772) was purchased from American Type Culture Collection (ATCC) (LGC Promochem GmbH, Germany) and routinely cultured at low density in Dulbecco's' modified Eagle's medium supplemented with 10% fetal calf serum, with serial passages by trypsination. The serum was pretested to ensure that it contained as little as possible osteogenic activity towards C2C12 [9]. For testing the extract, 1,000 cells/well were placed into 96-well microplates and allowed to adhere overnight. Medium was then exchanged with medium containing defined amounts of extract respectively recombinant human BMP-2 [10] and BMP-7 (Stryker Biotech, Hopkinton, MA, USA) standards, plus 10 μg/ml vitamin C and 20 nM vitamin D (cholecalciferol, Sigma-Aldrich, Munich, Germany). After 5 days of cultivation, the medium was removed and cells were washed once with PBS before determination of alkaline phosphatase (AP) activity, as a marker for osteogenic activation. To detect localized BMP activity in bursa tissue, frozen sections (10 μm) were dried onto sterile glass slides and 10,000 C2C12 cells were seeded onto each slide. Following 5 days of incubation, the cells were fixed with 4% paraformaldehyde in PBS and submitted to AP detection, as described below.

### Alkaline phosphatase

AP activity in 96-well C2C12 cultures was detected and quantified using an AP chemiluminescence detection kit (Roche Diagnostics GmbH, Mannheim, Germany). As a calibration, purified AP (activity 35,820 units/ml; EMD Biosciences, VWR DEUTSCHLAND GMBH, Darmstadt, Germany) was serially diluted and allowed to react with the kit in parallel wells without cells. Detection of bioluminescence was performed in a FluorS MultiImager (Bio-Rad Munich, Germany). Enzyme activity was evaluated against a calibration curve in the activity range from 1791 down to 17.91 μU (regression coefficient, >0.9) with purified bone AP. Significance (*P*) was calculated with a paired *t *test, comparing quadruplicates from the control group with each treatment group. For qualitative detection of AP activity in histological sections, frozen sections or paraffin-embedded sections (after deparaffination) were incubated with an NBT/BCIP detection kit (Roche Diagnostics GmbH, Mannheim, Germany), forming a dark blue precipitate. Levamisole (0.5 mM) was used, as directed by the manufacturer, to differentiate bone AP from other isoenzymes.

### RNA and reverse transcription-PCR

Reverse transcription-PCR (RT-PCR) was applied to detect mRNA of BMP, matrix proteins, and inflammatory cytokines (primers and conditions; Table 1). The primers were optimized for performance. Sequence specificity was tested by BLAST searches [11]. Total RNA was isolated from the TRIzol™ extract according to the manufacturer's protocol and stored at -80°C before use. Aliquots were submitted to reverse transcription, amplified by PCR, analyzed in 0.8% agarose gels, and normalized to glucosealdehyde phosphate dehydrogenase (GAPDH) and actin mRNA. The bands were visualized in a FluorS MultiImager.

The real-time quantitative RT-PCR reactions were carried out, using an iCycler 'iQ Real-Time PCR' instrument (Bio-Rad Munich, Germany) and an 'IQ SYBR Green Supermix' (Bio-Rad Munich, Germany), with primers that specifically amplified the transcripts of the genes of interest. The primers used for qRT-PCR were the same as those used for RT-PCR, with the exception of GAPDH, type II collagen, and tumor growth factor (TGF)-β mRNA-specific primer pairs. These were as follows (upstream and downstream primers, and expected product size (base pairs (bp))):for GAPDH, 5'-CATCACTGCCACCCAGAAGA-3', 5'-CCTGCTTCACCACCTTCTTG-3', and 254 bp (NCBI: NM_000660); for type II collagen, 5'-CAACACTGCCAACGTCCAGAT-3', 5'-CTGCTTCGTCCAGATAGGCAAT-3' [12], and 107 bp; for TGF-β, 5'-CGGCAGCTGTACATTGACTT-3', 5'-AGCGCACGATCATGTTGGAC-3', and 270 bp [NCBI: NM_002046]

**Figure 1:**
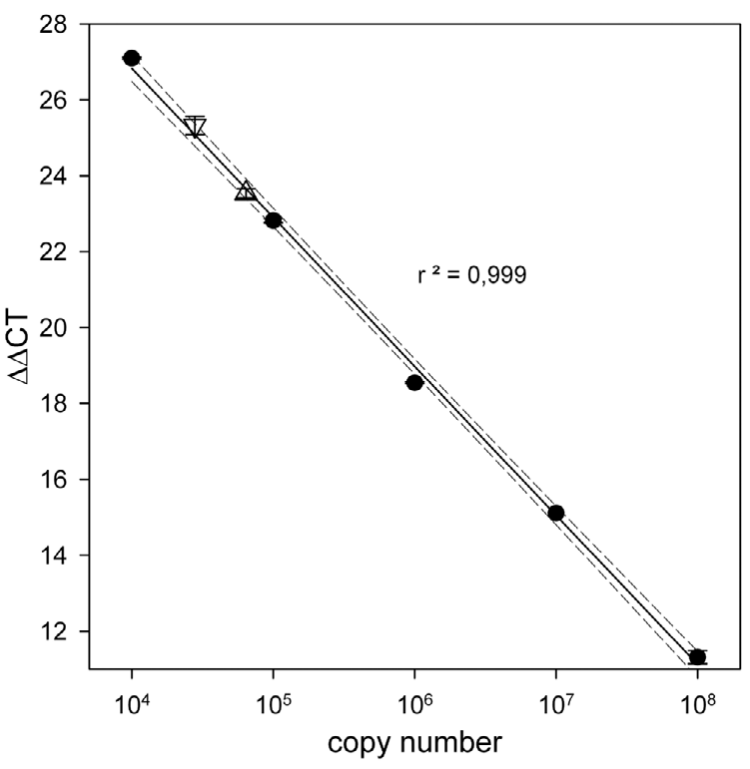
Example of a calibration experiment for the determination of the ΔΔCt values. The figure shows the standard curve for glucosealdehyde phosphate dehydrogenase (GAPDH) based on the plasmid used for standardization. The regression curve has the function f(x) = -3.9x + 42.5. The dashed lines indicate the 95% confidence intervals. Moreover, two aliquots were prepared containing cDNA from a bursa sample, in addition to the plasmid standard. Upright triangle: one volume of bursa sample. Inverted triangle: two volumes of bursa sample. Note that the linearity of measurement is undisturbed by the added samples and that the detected quantities of GAPDH strictly correlate to the amounts added.

An example of the standard curves obtained is given in Figure 1, including a calibration curve for an unknown sample. Each sample was measured in duplicates, with a 2.3% average error of measurement determined across all samples. Relative gene-expression levels were calculated as 2(ΔCt), with GAPDH used for normalization [12-15].

### Immunohistochemistry

Frozen sections (5 μm) were obtained from the deep-frozen bursae and stained by indirect immunofluorescence procedures. Mouse monoclonal antibodies were chosen for BMP-2/4 (final concentration, 2.5 μg/ml; MAB 355, R&D Systems,) and for BMP-7 (final concentration, 40 μg/ml; MAB 3541, R&D Systems, Wiesbaden.Nordenstadt, Germany). Parallel sections were stained to obtain information on approximate co-localization and BMP activity on application to C2C12 cells (see above).

### Statistical analysis and graphs

Statistical calculations were performed using SigmaStat 3.0 (SPSS GmbH, Munich, Germany). The graphs were made with SigmaPlot 8.0 (SPSS).

## Results

### Immunohistology and histochemistry

**Figure 2:**
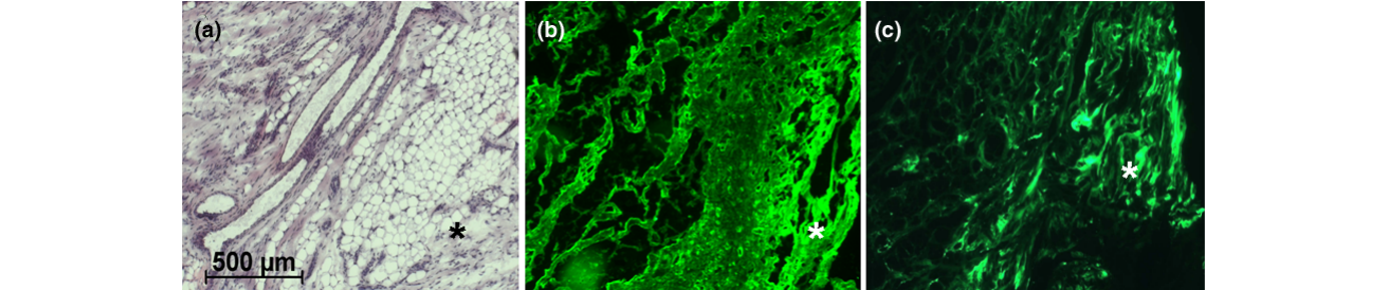
Hematoxylin-eosin staining **(a) **and indirect immunofluorescence for collagen types I **(b) **and II **(c) **in comparable areas. The acromial portion of the bursa is in the upper left-hand corner and the segment close to the rotator cuff is in the bottom right-hand corner, with some fat tissue in between. The asterisk (*) marks dense connective tissue typical of the transition zone between bursa and tendon tissue.

The overall morphology of the inflamed bursa has been described by others [6]. The top portion, close to the acromion, looks similar to synovial tissue with lining cells. The center comprises reticular connective tissue and fat tissue, with vessel structures that seem not only to be arterioles and venules, but also elements similar to interdigitations of the synovial lining. The bottom portion, close to the tendon, displays the morphology of dense connective tissue, with transition to a classical tendon-like structure. There were bursa tissues from patients with various degrees of degeneration if amounts of fat tissue and disorganized connective tissue were selected as pathologic signs. But with the absence from the literature of an established histological grading system for subacromial bursa pathology, we refrained from discriminating grades or stages. Collagen type I is present between the lobular elements in the supporting reticular tissue of the bursa and the supraspinatus tendon fibers reaching into the basal portion of the organ, in addition to the dense connective tissue between the bursa and the tendon (Figure 2b). Although we detected collagen type II mRNA in most specimens (see below), collagen type II protein was detected only in some cases and only with spotty distribution, preferentially towards or within the tendon. Elements staining positive included reticular fibers, dense connective tissue fibers proximal to the tendon, and the tendon (Figure 2c). Safranin O staining, an indicator of large deposits of proteoglycan, was negative (not shown). This is in agreement with the absence of mRNA for aggrecan in this tissue (see below).

**Figure 3:**
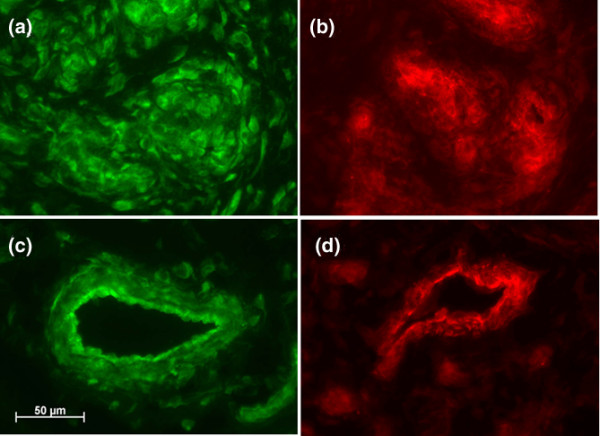
Indirect immunofluorescence images of bone morphogenetic proteins (BMP)-2 (green) and BMP-7 (red) in two areas of inflamed bursa. **(a) **and **(b) **are from cell-rich areas; **(c) **and **(d) **are microvessels. Objective magnification: ×40.

Within these structures, there were significant deposits of BMP-2 and BMP-7, as visualized by indirect immunofluorescence staining. The staining was concentrated in islets of high cellularity (Figure 3a,b), vessel walls (Figure 3c,d), and segmentally arranged lining cells (see below) rather than along connective tissue fiber structures. The dense connective tissue and tendon were mostly negative.

**Figure 4:**
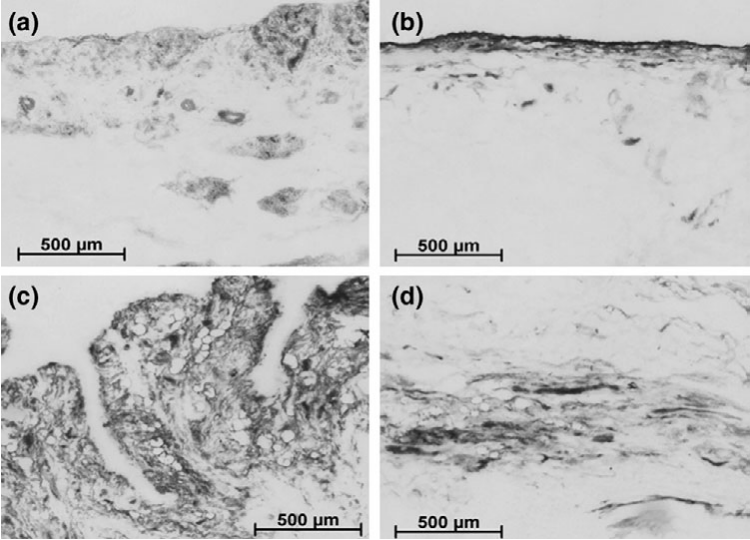
Alkaline phosphatase activity within the subacromial bursa varies from patient to patient. Some activity is located within the lining and underlying 'lobules' **(a) **and **(b)**, in addition to within the connective tissue of the bursa **(c) **or the border between bursa and tendon tissue **(d)**. In all cases, treatment of the sections with 0.5 mM levamisole eradicated the enzyme reaction, leaving behind only staining in granules within individual cells that appeared to be leukocytes (not shown).

Bone AP, which could be inhibited by 5 mM levamisole to differentiate it from leukocyte AP, was detected preferentially within the bursa, not the tendon, and always close to vessel or duct structures and the acinar elements in the acromial portion (Figure 4).

### Expression of mRNA for bone morphogenetic protein, matrix proteins, and cytokines

**Figure 5:**
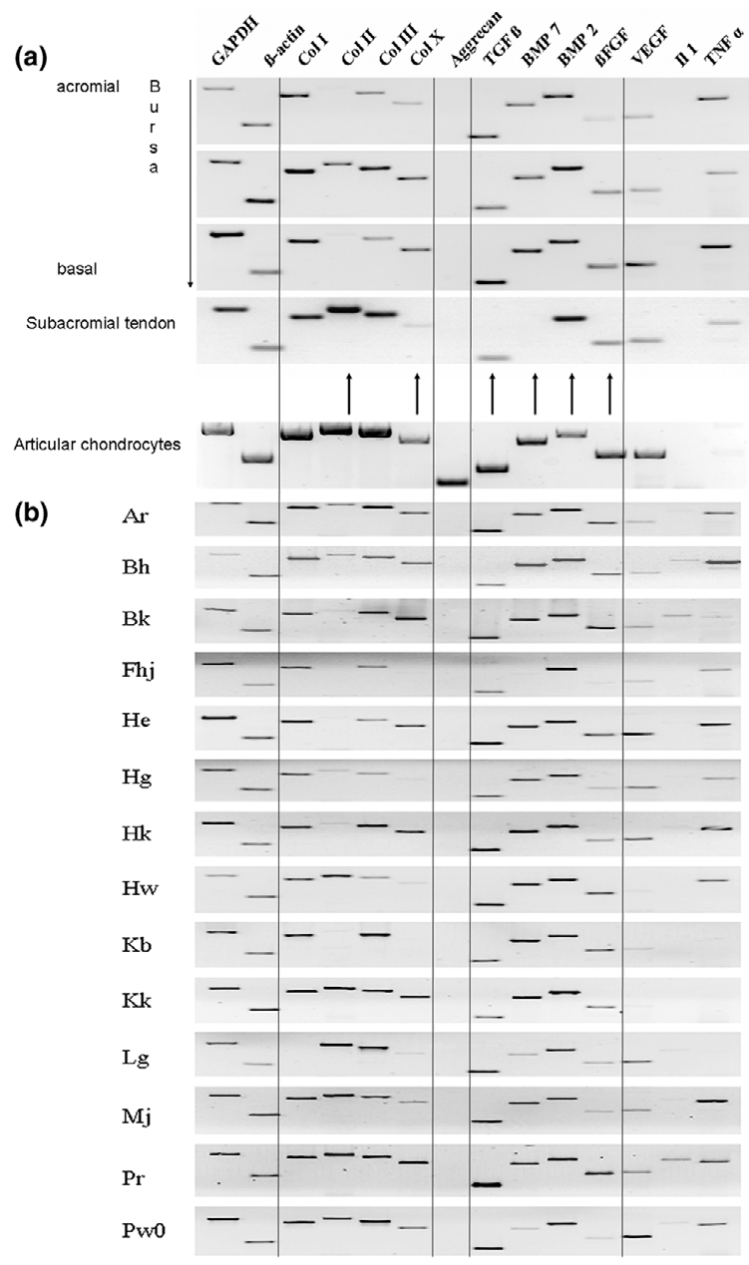
A comparison of mRNA profiles for select genes from bursa tissue, tendon and cartilage. **(a) **Gel-electrophoretic profile of reverse transcription (RT)-PCR from RNA of a subacromial bursa and the underlying torn supraspinatus tendon. Ethidium bromide-stained electrophoresis gels are shown; the image grey scale has been inverted. The samples have been normalized for total RNA extracted from the tissue, and GAPDH and actin are presented as standards. Tissue aliquots were sampled anatomically. The dark arrows indicate mRNA that have been quantified by real-time PCR (Figure 4). An example from cultured human articular chondrocytes has been added for comparison. Note the absence of mRNA from the cartilage proteoglycan aggrecan in the bursa specimens. **(b) **– Gel-electrophoretic RT-PCR profile of subacromial bursa RNA from 14 patients. Note that the level of IL-1β mRNA is weak or absent in most samples from the bursa; TNF-α is absent in some bursa specimens. BMP, bone morphogenetic protein; COL, collagen type; bFGF, basic fibroblast growth factor; GAPDH, glucosealdehyde phosphate dehydrogenase; IL, interleukin; TGF, tumor growth factor; TNF, tumor necrosis factor; VEGF, vascular endothelial growth factor.

**Figure 6:**
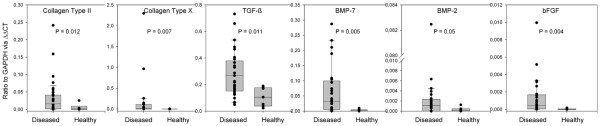
Expression levels of cartilage-specific mRNA and growth factor mRNA types from seven diseased and two healthy bursa specimens. Each patient is represented by four measurements (dots in the graph) across the bursa, which are derived from the acromial, medial, basal, and tendineous portions of the organ. The box plots are made up of all the combined values. All data sets are statistically significant, with *P *< 0.05. ΔΔCt, relative mRNA expression levels; BMP, bone morphogenetic protein; bFGF, basic fibroblast growth factor; GAPDH, glucosealdehyde phosphate dehydrogenase; TGF, tumor growth factor.

Figures 5 and 6 show the results of expression profiles obtained from areas of the bursa that are proximal to the acromion, central, proximal to the tendon, and underlying the supraspinatus tendon segment. As expected, mRNA for collagen types I and III are present in all samples. Collagen type II, the major collagen type in chondrocytes, is significantly expressed within the bursa and underlying tendineus tissue of samples from almost all patients (Figures 5 and 6), possibly a sign of the ongoing transformation of tendon to cartilage. The ratio of collagen type II to GAPDH mRNA in diseased tissue is less than or equal to 0.2, whereas in healthy tissue it is 0.02. By contrast, freshly isolated adult articular chondrocytes can have a collagen type II/GAPDH mRNA ratio of up to 100 (unpublished data). Interestingly, collagen type X expression (Figures 5 and 6) within the bursa is higher than in the tendon (ratios of collagen type X mRNA to GAPDH mRNA of less than or equal to 0.3 and 0.04, respectively); such ratios in adult cartilage (Mollenhauer JA, unpublished data) are not different from those found in the diseased bursa tissue. Collagen type X is usually expressed in hypertrophic cartilage and is associated with mineralization processes; it is contained in mineralizing vesicles. In all of the samples tested there was no mRNA for the large cartilage proteoglycan aggrecan. However, in RNA prepared from articular cartilage, aggrecan mRNA was strongly displayed using the same RT-PCR conditions (Figure 5).

mRNA for growth factors of the TGF-β superfamily was found in all samples (some stratification was typical), with the following order of magnitude: TGF-β (in a ratio to GAPDH of 0.3) > BMP-7 (in a ratio to GAPDH of 0.03) > BMP-2 (in a ratio to GAPDH of 0.001). BMP-2 did not show preferential sublocation in its expression; however, BMP-7 mRNA in the acromial portion of the diseased bursa was approximately two orders of magnitude higher than in the healthy samples (in a ratio to GAPDH of 0.09 compared with 0.0009, respectively), representing the largest difference in growth factor expression between diseased and healthy tissues.

As examples of alternative tissue growth factors, fibroblast growth factor (FGF)-2 and vascular endothelial growth factor (VEGF) mRNA was also present and helps to explain the proliferation of the bursa into granulation tissue, with loose connective tissue and blood vessels amply present. However, FGF-2 mRNA levels were low throughout the tissue – the ratio of FGF-2 mRNA to GAPDH mRNA was about 10 times lower than the ratio of BMP mRNA to GAPDH mRNA- and was not preferentially localized within the bursa. Of the family of inflammatory cytokines, we tested for interleukin (IL)-1 and tumor necrosis factor (TNFα. As in the present example, typically IL-1β mRNA was not prominent, whereas TNF-α mRNA was always expressed, in particular within the top portion of the bursa. Figure 5b shows a representative cross-sectional mRNA analysis from 14 patients.

### Biogenic bone morphogenetic protein activity

We decided on a bioassay for differentiation rather than directly determining quantities of specific BMPs. Although measuring BMP concentrations is an exact method, no information is given on the bioactivity, because molecules might exist in an inactive pro-form, partially degraded or denatured, or in mixtures that cancel out effects on differentiation. The C2C12 cell line is an established detector of BMP type I receptor-related BMP activity owing to its induction of AP [9]. On the basis of this receptor constellation, BMP-2 and BMP-4 should be initiators of osteogenic differentiation. However, we also found, although to a lesser degree, that BMP-7 could induce AP within these cells. In the extract, we found that the BMP equivalent induced 380 ± 135 μU AP/100 mg of bursa tissue, with the medium control displaying 175 ± 51 μU AP/100 mg of bursa tissue (*P *= 0.0163). By contrast, under the same conditions, 25 ng/ml recombinant BMP-2 induced 460 ± 154 μU AP/100 mg of bursa tissue (*P *= 0.0021) and 50 ng/ml of recombinant BMP-7 induced 430 ± 111 μU AP/100 mg of bursa tissue (*P *= 0.0058). BMP activity could be completely abolished by incubation with neutralizing antibodies against BMP-2 and BMP-7, in addition to incubation with the soluble BMP receptors Alk-3 and Alk-6 (not shown).

**Figure 7:**
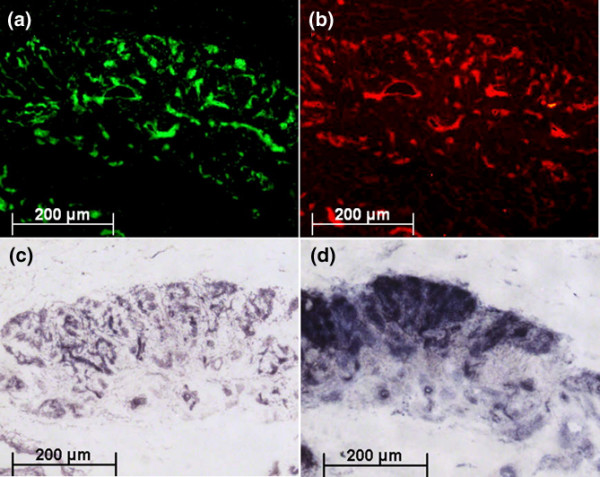
BMP-2 and BMP-7 co-localization with AP in bursa tissue with and without C2C12 cells. Indirect immunofluorescence staining of the topical distribution of **(a) **bone morphogenetic protein (BMP)-2 (green) and **(b) **BMP-7 (red) and their local effects on C2C12 cells. The images display objective magnifications (×5) of subsequent frozen sections. **(c) **A section without cultured C2C12 cells stained directly for alkaline phosphatase (AP). **(d) **A section seeded with C2C12 cells and cultured for 5 days before staining for AP. Note the co-distribution of AP, both the bursa-derived intrinsic enzyme and the C2C12-derived enzyme, with the BMP deposits within the tissue.

Because the extraction process does not enable determination of the original site of expression within the bursa tissue, we tested frozen sections of bursa tissue for their ability to induce AP locally in C2C12 cells seeded onto a frozen section. Surprisingly, the cells not only started to express AP, but also expression was confined to the location where we detected BMP-2 and BMP-7 in parallel sections (Figure 7). No activation of AP expression took place distant from those sites. Although the microwell assay worked well with the BMP extract (BMP in solution), the result from the 'contact' experiment indicates that BMP might also exert its effect strictly locally, thus avoiding lateral expansion of the differentiation signal.

## Discussion

Degenerative alterations to the rotator cuff are generally regarded as the consequence of mechanical abutment, undefined 'rheumatoid' inflammation, or previous accident. Although these causes could describe possible etiologies, they do not completely explain the cellular and molecular alterations seen in and around the rotator cuff, such as chondrogenic transformation and ectopic mineralization of the tendon tissue. With the present set of data, an additional perspective towards a causative explanation is given: chronic activation of morphogenetic factors (BMP-2, BMP-7, TGF-β, VEGF, and FGF) that might actively contribute to the rise of mechanically incompetent and (because of the mineral deposits) chronically irritating tissue components. The differential expression of BMP-2 and BMP-7, with BMP-7 expressed in a decreasing gradient from acromion to tendon, suggests a distinct contribution of BMP-7 to the disease process. Additional expression of inflammatory cytokines (IL-1β and TNF-α) might serve in propagating local inflammation and tissue destruction.

In bursa samples from patients with ruptures of the rotator cuff, expression of collagen types I and III was enhanced compared with normal controls [16]. In addition, enhanced expression of IL-1β, both secreted and cell-bound IL-1 receptor antagonists, and VEGF have been demonstrated [4,17,18]. Participation of the bursa in the disease progression of the rotator cuff has also been shown in animal experiments with rabbits [19] and chickens [20]. Specifically, chemical induction of a bursitis-induced chondrogenic metaplasia of the supraspinatus insertion site [21]. These reports are not only in line with our findings, but also strongly suggest significant influence of molecular events within the subacromial bursa on the fate of the underlying supraspinatus tissue.

Unfortunately, there is no recent literature on the histology of the normal bursa. Organ material from tumor-related and joint-replacement surgery was available but always showed signs of degeneration, depending on the primary disease, and was thus unrepresentative of a normal situation. Nonetheless, the presence of bioactive BMP in itself supports the hypothesis of its role in induced chondrogenesis, irrespective of whether and, if so, how much it is expressed in normal tissue.

The amounts of BMP we detected are quite significant. Although direct estimates are hard to compare, the mid-ng quantities/mg of bursa tissue represent an overwhelmingly strong potential for morphogenetic signaling. In particular, the in-situ transformation that was achieved by placing the detector cell line C2C12 onto tissue slices reveals the effectiveness of the deposited BMP within the bursa. BMP detected within the cell and extracellular matrix of blood vessels might have been transported there through the bloodstream. However, the microanatomic distribution of immunohistological signals and the results from the PCR analysis strongly suggest local production rather than introduction from outside the bursa. Because we could block the differentiation signal by incubating the extracts with anti-BMP antibodies or soluble BMP receptors, a dominant role of BMPs in activating tissue transdifferentiation can be assumed, although a contribution of other growth factors cannot be excluded.

Because of the normal function of the bursa, the BMP might reach the tendon tissue through anatomic secretion pathways from the glandular elements within the bursa. Unfortunately, too little is reported in the literature about the secretary activity of this normally rather inconspicuous layer of tissue underneath the acromion. It is obvious, however, that there was significant cartilage differentiation, with RNA levels for cartilage collagen types II and X at quite significant levels in parts of the patients' tissue. Collagen to GAPDH ratios of >0.1, as detected here, are usually found in normal articular, mineralizing, and osteoarthritic tissue [22,23]. In addition, studies performed in the rat support our observation of long-term preservation of cartilage-related gene expression in the supraspinatus [24].

There is some information on the production and role of BMP in adult soft tissue. BMP is produced by gingival and periodontal fibroblasts [25], megakaryocytes and platelets [26], cells supporting egg maturation [27,28], the kidney [29], and also connective tissue tumor cells. More importantly, arthritic synovial membranes have been shown to express BMP-2 and BMP-6 and can influence cell turnover [30]. Early studies showed that BMP induces tissue transdifferentiation of tenocytes into chondrocytes *in vitro *[31] and, more recently, studies showed BMP-induced transdifferentiation of kidney fibroblasts into epithelial cells [32]. Our finding in itself is not unexpected, retrospectively, but so far, to our knowledge, no attempts have been made to directly link the 'shoulder syndrome' to ectopic overexpression of BMP. Our approach using AP differentiation within the C2C12 cell line gave us a tool to explore primary features of the BMP deposited within the bursa. However, to describe the entire pathologic pathway from soft tissue to mineral deposits and fiber cartilage, more experiments are needed using chondrocytes and primary mesenchymal precursor cells *in vitro *or with exogenous deposits of defined BMP in animals.

## Conclusion

In the absence of pharmacologic strategies to counteract untoward BMP activity, only surgical intervention is an option for curative approaches to preventing eventually dramatic outcomes, such as in cases of ossification of the rotator cuff [33]. Complete excision of the subacromial bursa either before acute rupture or during restorative surgery of torn ligaments or the tendon might represent the only option we currently have.

## Abbreviations

AP = alkaline phosphatase; BMP = bone morphogenetic protein; ΔCt = threshold cycle number for amplified cDNA; FGF = fibroblast growth factor;

GAPDH = glyceroaldehydephosphate dehydrogenase; IL = interleukin; PBS = phosphate-buffered saline; RT-PCR, reverse transcription-PCR; TGF = tumor growth factor; TNF, tumor necrosis factor; VEGF = vascular endothelial growth factor.

## Competing interests

The authors declare that they have no competing interests.

## Authors' contributions

RF, JN, AV, and IS contributed equally to the preparation of the manuscript.

## Figures and Tables

**Table 1:** Primers for RT-PCR

**Gene**	**Primer**	**Sequence**	**RT-PCR product (base pairs)**	**Annealing temperature**	**Cycles**	**Reference**
GAPDH	up	CCACCCATGGCAAATTCCATGGCA	600	60	25	Stratagene
	down	TCTAGACGGCAGGTCAGGTCCACC				
Actin	up	TGAAGTCTGACGTGGACATC	254	60	25	[7]
	down	ACTCGTCATACTCCTGCTTG				
COL1A2	up	AGACCCAAGGACTATGAAGT	509	55	25	[NCBI: NM_000089]
	down	ACATCATTAGAGCCCTGTAG				
COL2A1	up	CATCTGGTTTGGAGAAACCATC	606	60	37	[NCBI: J00116]
	down	GCCCAGTTCAGGTCTCTTAG				
COL3A1	up	GATGGGGTCAAATGAAGGTGA	546	60	25	[NCBI: NM_000090]
	down	GCAGATGGGCTAGGATTCAAA				
COL10A1	up	CCTCTTGTTAGTGCCAACCAG	424	60	37	[NCBI: X98568]
	down	GAGCCACTAGGAATCCTGAG				
Aggrecan	up	ACTTCCGCTGGTCAGATGGA	111	55	37	[8]
	down	TCTCGTGCCAGATCATCACC				
TGF-β1	up	CAGAAATACAGCAACAATTCCTGG	187	60	37	Stratagene
	down	TTGCAGTGTGTTATCCGTGCTGTC				
BMP-7	up	TTTGGGGCCAAGTTTTTCTG	410	60	37	[9]
	down	ACAGGAACTTCCGGGTCAAT				
BMP-2	up	GGGAAAACAACCCGGAGATT	503	60	37	[NCBI: NM_001200]
	down	TTAAGGCGTTTCCGCTGTTT				
FGF-2	up	TACAACTTCAAGCAGAAGAG	283	60	37	[10]
	down	CAGCTCTTAGCAGACATTGG				
VEGF	up	AAGTGGTCCCAGGCTGCA	296	60	37	[NCBI: AY047581]
	down	ATCTCTCCTATGTGCTGGCC				
IL-1	up	AAGCAGCCATGGCAGAAGTA	482	60	37	[NCBI: M15840]
	down	GAACACCACTTGTTGCTCCA				
TNF-	up	GAGTGACAAGCCTGTAGCCCATGTTGTAGCA	444	60	37	Clontech
	down	GCAATGATCCCAAAGTAGACCTGCCCAGACT				

The sources for sequences are given in the last column, the numbers in brackets refer to the literature, and the other numbers indicate the codes from sequences in PubMed. BMP, bone morphogenetic protein; COL, collagen type; FGF, fibroblast growth factor; GAPDH, glucosealdehyde phosphate dehydrogenase; IL, interleukin; RT-PCR, reverse transcription-PCR; TNF, tumor necrosis factor; VEGF, vascular endothelial growth factor.
